# Intimal lining layer macrophages but not synovial sublining macrophages display an IL-10 polarized-like phenotype in chronic synovitis

**DOI:** 10.1186/ar3796

**Published:** 2012-04-11

**Authors:** Carmen A Ambarus, Troy Noordenbos, Maria JH de Hair, Paul P Tak, Dominique LP Baeten

**Affiliations:** 1 Department of Clinical Immunology and Rheumatology, Academic Medical Center/University of Amsterdam, Meibergdreef 9, 1105 AZ Amsterdam, The Netherlands; 2 Department of Experimental Immunology, University of Amsterdam, Meibergdreef 9, 1105 AZ Amsterdam, The Netherlands; 3 GlaxoSmithKline, Gunnels Wood Road, Stevenage Herts, SG1 2NY, UK

## Abstract

**Introduction:**

Synovial tissue macrophages play a key role in chronic inflammatory arthritis, but the contribution of different macrophage subsets in this process remains largely unknown. The main *in vitro *polarized macrophage subsets are classically (M1) and alternatively (M2) activated macrophages, the latter comprising interleukin (IL)-4 and IL-10 polarized cells. Here, we aimed to evaluate the polarization status of synovial macrophages in spondyloarthritis (SpA) and rheumatoid arthritis (RA).

**Methods:**

Expression of polarization markers on synovial macrophages, peripheral blood monocytes, and *in vitro *polarized monocyte-derived macrophages from SpA versus RA patients was assessed by immunohistochemistry and flow cytometry, respectively. The polarization status of the intimal lining layer and the synovial sublining macrophages was assessed by double immunofluorescence staining.

**Results:**

The expression of the IL-10 polarization marker cluster of differentiation 163 (CD163) was increased in SpA compared with RA intimal lining layer, but no differences were found in other M1 and M2 markers between the diseases. Furthermore, no significant phenotypic differences in monocytes and *in vitro *polarized monocyte-derived macrophages were seen between SpA, RA, and healthy controls, indicating that the differential CD163 expression does not reflect a preferential M2 polarization in SpA. More detailed analysis of intimal lining layer macrophages revealed a strong co-expression of the IL-10 polarization markers CD163 and cluster of differentiation 32 (CD32) but not any of the other markers in both SpA and RA. In contrast, synovial sublining macrophages had a more heterogeneous phenotype, with a majority of cells co-expressing M1 and M2 markers.

**Conclusions:**

The intimal lining layer but not synovial sublining macrophages display an IL-10 polarized-like phenotype, with increased CD163 expression in SpA versus RA synovitis. These differences in the distribution of the polarized macrophage subset may contribute to the outcome of chronic synovitis.

## Introduction

The normal synovial membrane consists of two distinct structures: the intimal lining layer and the synovial sublining. The normal intimal lining layer comprises fibroblast-like synoviocytes and intimal lining layer macrophages, whereas the synovial sublining consists of connective tissue containing blood vessels, fibroblasts, adipocytes, and a limited number of resident immune cells, such as macrophages and mast cells [1]. Chronic inflammatory arthritis is histologically characterized by marked hyperplasia of the intimal lining layer and massive infiltration of the synovial sublining with innate and adaptive immune cells, which release inflammatory mediators, promote neoangiogenesis, and drive the destruction of adjacent cartilage and bone [2]. Macrophages play a major role in this process because they contribute to the intimal lining layer hyperplasia [3] and are the main producers of key inflammatory mediators, such as tumor necrosis factor (TNF) [4]. Accordingly, the number of synovial macrophages correlates with clinical disease activity [2,5] and decreases after clinically efficient but not placebo treatment in both rheumatoid arthritis (RA) [6-9] and spondyloarthritis (SpA) [10-13]. Furthermore, selective macrophage depletion has a strong antiinflammatory effect in animal models of arthritis [14].

Tissue macrophages consist of resident macrophages and monocytes that are recruited in inflammatory conditions and differentiate into macrophages on tissue entry. These macrophages represent a heterogeneous population, as local mediators can prime macrophages during their maturation and thereby shape their subsequent response to various activating stimuli [15,16]. Initially, two main polarized macrophage subsets were described based on their pro- versus antiinflammatory functions: classically activated macrophages (M1), which are specialized in the clearance of intracellular pathogens, and alternatively activated macrophages (M2), which have immunoregulatory properties and are involved in scavenging debris, angiogenesis, and tissue repair [17]. The M1 subset is induced mainly by interferon (IFN)-γ (MΦ_IFN-γ_), whereas M2 can be induced *in vitro *either by IL-4 (MΦ_IL-4_)/IL-13 or by IL-10 (MΦ_IL-10_) [17-20]. Whereas the macrophage polarization has been best described in rodents, we recently validated specific phenotypic markers [21] and distinct functional characteristics (unpublished observations) for these three main *in vitro *polarized macrophage subsets in humans.

The increasing knowledge of the phenotypic and functional diversity of human macrophages indicates that not only the overall number of macrophages in the inflamed tissue, but also their specific polarization status may determine the disease outcome in terms of severity, chronicity, and tissue damage. Interestingly, we previously described that, despite similar numbers of macrophages in RA and SpA synovitis, a selective overrepresentation of CD163^+ ^macrophages was present in SpA [3,22-25]. CD163, the scavenger receptor for hemoglobin/haptoglobin complexes, is a known marker for IL-10 or M-CSF polarized M2 [21,26]. Together with the lower levels of M1-derived proinflammatory cytokines in SpA versus RA synovial fluid [27] and the defective IFN-γ signature in SpA monocytes [28], these data suggest a preferential M2 polarization in SpA compared with an M1 polarization in RA. The aim of this study was to test this hypothesis by detailed characterization of distinct macrophage subsets in SpA and RA synovial tissue.

## Materials and methods

### Patients and samples

Peripheral blood samples were obtained from 11 SpA and eight RA patients and nine healthy controls. Synovial biopsies were obtained with small-bore arthroscopy from clinically inflamed knee joints, as previously described [29], and 18 SpA and 20 RA patients were included for the immunohistochemical analysis. All SpA patients fulfilled the criteria of the European Spondyloarthropathy Study Group (ESSG) [30], and all RA patients fulfilled the 1987 ACR classification criteria for RA [31]. Demographic and clinical data of the patients are shown in Table 1. None of the patients was being treated with biologicals. All patients gave written informed consent to participate to the study, as approved by the Medical Ethics Committee of the Academic Medical Centre/University of Amsterdam.

**Table 1:** Demographic and clinical features of the patients in the two cohorts

	Cohort 1		Cohort 2	
	
	SpA (*n *= 18)	RA (*n *= 20)	SpA (*n *= 11)	RA (*n *= 8)
Gender, % female	40	58	33	100

Age, years	47 (33-55)	62 (53-74)	38 (31-44)	48 (32-63)

Disease duration, years	3 (1-29)	3 (1-11)	2 (1-4)	2 (1-5)

Swollen-joint count	1 (1-2)	8 (5-15)	1 (0-1)	1 (0-2)

CRP, mg/L	12 (5-31)	19 (8-58)	2 (1-7)	5 (4-11)

ESR, mm/hour	29 (23-43)	47 (33-66)	2 (2-16)	21 (13-29)

% using DMARDs	80	54	50	100

As described in Materials and methods, synovial tissue biopsies were obtained from patients with spondyloarthritis (SpA) and rheumatoid arthritis (RA) (Cohort 1). Monocytes were isolated from peripheral blood of SpA and RA patients (Cohort 2). Except when indicated otherwise, values are expressed as the median (interquartile range). CRP, C-reactive protein; DMARD, disease-modifying anti-rheumatic drug; ESR, erythrocyte sedimentation rate.

### Immunohistochemistry

Synovial biopsy samples (six to eight per patient to minimize sampling error) were snap-frozen *en bloc *and mounted in Jung tissue-freezing medium (Leica Instruments, Nussloch, Germany). The acetone-fixed sections were stained by using mouse monoclonal antibodies directed toward CD68 (clone EBM-11; Dako, Heverlee, The Netherlands), CD64 (clone 10.1; BioLegend, Uithoorn, The Netherlands), CD14 (clone TUK4; Dako), CD163 (clone 5cFAT; BMA Biomedicals, Augst, Switzerland), and CD32 (clone AT10; abcam, Cambridge, UK). Sections were sequentially incubated with a biotinylated secondary antibody, a streptavidin-horseradish peroxidase link (LSAB; Dako), aminoethylcarbazole substrate as chromogen, and hematoxylin as counterstain [22,25]. Parallel sections were incubated with isotype- and concentration-matched monoclonal antibodies as negative controls. The expression of CD200R was assessed with immunofluorescence, by using a mouse monoclonal antibody (clone OX108; AbD Serotec, Düsseldorf, Germany). For each cohort, samples were stained in a single run, coded, and scored in a random order on a 4-point semiquantitative scale by two independent observers (CAA, TN), as described previously [5].

### Monocyte isolation and *in vitro *polarization

Monocytes were isolated from peripheral blood with gradient centrifugation with Lymphoprep (Axis-Shield PoPAS, Oslo, Norway) and, subsequently, Percoll gradient separation (GE Healthcare, Uppsala, Sweden). Monocytes were analyzed immediately with flow cytometry or cultured at a concentration of 0.5 × 10^6^/ml in Iscove Modified Dulbecco Medium (IMDM) (Invitrogen, Breda, The Netherlands) supplemented with 10% fetal calf serum (FCS) (PAA Laboratories, Cölbe, Germany). Cultured monocytes were polarized *in vitro *with human recombinant IFN-γ (10 ng/ml; R&D Systems, Abingdon, UK), IL-4 (10 ng/ml; Miltenyi Biotec, Bergisch Gladbach, Germany), or IL-10 (10 ng/ml; R&D Systems) for 4 days. For further analysis, polarized macrophages were recovered by scraping of the culture plate.

### Flow cytometry

Surface-marker expression on monocytes and *in vitro *polarized macrophages was analyzed with flow cytometry (BD FACS Calibur Flow Cytometer, Erembodegem, Belgium) by using fluorochrome-labeled monoclonal antibodies against CD80 (clone L307.4; BD Pharmingen, Breda, the Netherlands), CD64 (clone 10.1; BioLegend), CD200R (clone OX108; AbD Serotec), CD14 (clone 61D3; eBioscience, San Diego, CA, USA), CD163 (clone GHI/61; BD Pharmingen), and CD16 (clone DJ130c; AbD Serotec, Düsseldorf, Germany). Equivalent amounts of isotype-matched control antibodies were included in all experiments as negative controls. Before staining, Fc receptors were blocked with 10% human serum (Lonza, Cologne, Germany). Data were analyzed with Flow Jo Flow Cytometry Analysis software (Tree Star, Ashland, OR, USA) after gating on the myeloid population in the FSC/SSC window. Values were expressed as the ratio of the geometric mean fluorescence intensity (gMFI) of the marker of interest over the gMFI of the isotype control.

### Double immunofluorescence

Frozen synovial tissue sections were fixed in acetone and blocked with 10% goat serum (Dako, Glostrup, Denmark), followed by incubation with Biotin blocking system (Dako). Mouse monoclonal antibodies against CD80 (clone 2D10; BioLegend, Uithoorn, The Netherlands), CD64 (clone 10.1; BioLegend), CD200R (clone OX108; AbD Serotec, Düsseldorf, Germany), CD14 (clone TUK4; Dako), CD163 (clone 5cFAT; BMA Biomedicals, Augst, Switzerland), CD16 (clone HI16a; Abbiotec, San Diego, CA, USA), and CD32 (clone AT10; abcam, Cambridge, UK) were added, followed by incubation with Alexa-555-conjugated goat anti-mouse antibody (Molecular Probes Europe, Leiden, The Netherlands). After blocking with 10% mouse serum (Dako), the sections were incubated with biotinylated mouse monoclonal antibodies against CD68 (clone Y1/82A; BioLegend), CD64 (clone 10.1; BioLegend), CD200R (clone OX108; AbD Serotec), and CD163 (clone GHI/61; BioLegend). After incubation with streptavidin-Alexa 488 (Molecular Probes Europe, Leiden, The Netherlands), the slides were mounted with Vectashield containing DAPI (Vector Laboratories, Burlingame, CA, USA) and analyzed on a fluorescent imaging microscope (Leica DMRA, Wetzlar, Germany) coupled to a charge-coupled device (CCD) camera and Image-Pro Plus software (Media Cybernetics, Dutch Vision Components, Breda, The Netherlands). To quantify the coexpression of CD68 with CD64, CD200R, CD14, and CD163, the number of positively stained cells was counted in a minimum of five microscopic fields, and the percentage of double-positive cells from the total number of CD68-positive cells was calculated.

### Statistics

Statistical analysis was performed by using Prism software (GraphPad, La Jolla, CA, USA). Data were expressed as median ± interquartile range. A Mann-Whitney test was used for comparisons between two groups (SpA compared with RA) and ANOVA followed by a Bonferroni posttest was used for comparisons among more than two groups. A *P *value of less than 0.05 was considered statistically significant.

## Results

### The expression of CD163 but not other M2 phenotypic markers is increased on intimal lining layer macrophages in SpA versus RA synovitis

**Figure 1:**
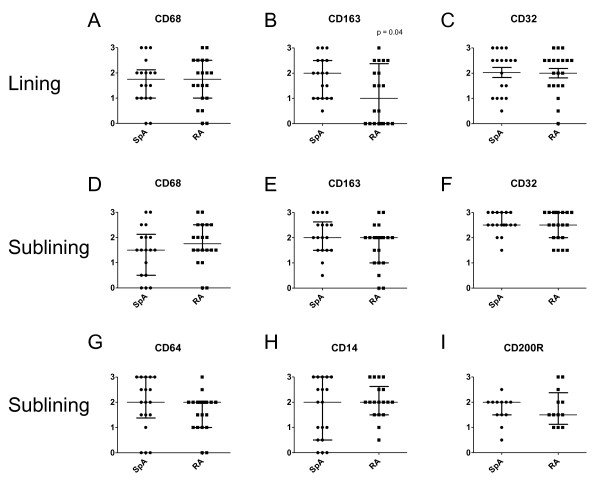
**Immunohistochemical analysis of the expression of polarization markers in spondyloarthritis (SpA) compared with rheumatoid arthritis (RA) synovial tissue**. The expression of CD68, CD163, and CD32 in the intimal lining layer **(A through C) **and the expression of CD68, CD64, CD200R, CD14, CD163 and CD32 in the synovial sublining **(D through I) **as assessed with semiquantitative scoring (0 to 3) of immunohistochemical stainings. Data are represented as median and interquartile range of 18 SpA and 20 RA patients.

To confirm and extend our previous description of increased CD163 expression in SpA compared with RA synovitis [3,22-25], we performed a detailed histologic analysis of the recently described panel of phenotypic markers for MΦ_IFN-γ_, MΦ_IL-4_, and MΦ_IL-10 _[21]. As previously shown, the number of CD68^+ ^macrophages in the intimal lining layer and synovial sublining was similar between the two diseases (Figure 1A, D), whereas CD163 expression was higher in the SpA compared with the RA intimal lining layer (*P *< 0.05) (Figure 1B), but similar in the synovial sublining (Figure 1E). However, the expression of another MΦ_IL-10 _marker, CD32, was similar in both diseases, for both the intimal lining layer and the synovial sublining (Figure 1C, F). The expression of the MΦ_IFN-γ _marker CD64, the positive MΦ_IL-4 _marker CD200R, and the negative MΦ_IL-4 _marker CD14 was similar in the synovial sublining of SpA and RA (Figure 1G through 1I) and was very low to absent in the intimal lining layer (data not shown). These histologic data confirmed the higher CD163 expression in SpA compared with RA synovitis, but failed to provide additional evidence for a biased M2 polarization in SpA.

### The phenotype of peripheral blood monocytes is similar in SpA and RA

**Figure 2:**
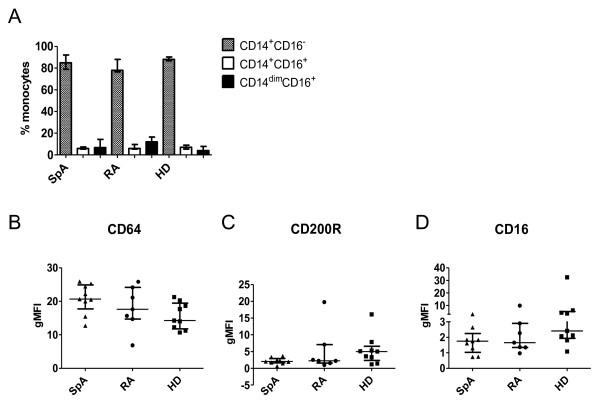
**Monocyte subsets and expression of phenotypic markers on spondyloarthritis (SpA) and rheumatoid arthritis (RA) patients and healthy donor monocytes**. The percentage of CD14^+^CD16^-^, CD14^+^CD16^+^, and CD14^dim^CD16^+ ^monocytes from SpA and RA patients and healthy donors was measured with flow cytometry **(A)**. Expression of CD64, CD200R, and CD16 on monocytes from SpA and RA patients and healthy donors was measured with flow cytometry and expressed as geometric mean fluorescence intensity (gMFI). Data are represented as median and interquartile range of at least seven independent experiments **(B)**.

To investigate whether the increased expression of CD163 in SpA synovitis is related to preferential MΦ_IL-10 _polarization, we set up a series of additional experiments. Because monocytes from peripheral blood of SpA patients display a defective IFN-γ signature [28] and IFN-γ is the prototypic M1 polarizing cytokine, we first assessed whether not only intimal lining layer macrophages, but also peripheral blood monocytes have a distinct phenotype in SpA. We quantified the percentage of classic CD14^+^CD16^- ^monocytes, intermediate CD14^+^CD16^+ ^monocytes, and nonclassic CD14^dim^CD16^+ ^monocytes [32] in peripheral blood from SpA and RA patients and healthy donors, and did not observe differences in these monocyte subsets between the groups (Figure 2A). We next assessed the expression of macrophage polarization markers on CD14^+ ^monocytes and measured a marked expression of the MΦ_IFN-γ _marker CD64 and a more modest expression of the MΦ_IL-4 _marker CD200R and the MΦ_IL-10 _marker CD16 (Figure 2B-D). However, no differences were found in the expression of these markers between SpA and RA patients and healthy controls. Expression of MΦ_IFN-γ _marker CD80 and MΦ_IL-10 _marker CD163 on monocytes was very low (data not shown). Based on this assessment, no indication exists of phenotypic differences between monocytes from SpA and RA patients and healthy individuals.

### The phenotype of *in vitro *polarized monocyte-derived macrophages is similar in SpA and RA

**Figure 3:**
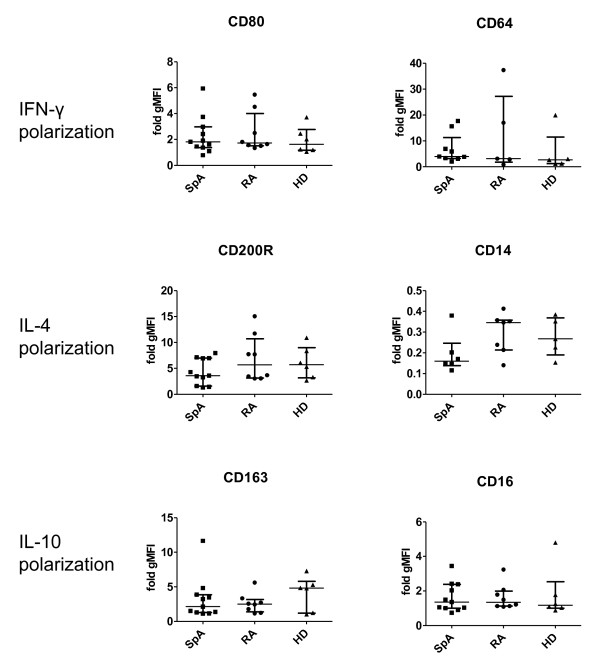
**Expression of phenotypic markers on *in vitro *polarized monocyte-derived macrophages from spondyloarthritis (SpA) and rheumatoid arthritis (RA) patients and healthy individuals**. The expression of MΦ_IFN-γ _markers CD80 and CD64, MΦ_IL-4 _markers CD200R and CD14, and MΦ_IL-10 _markers CD163 and CD16 was measured with flow cytometry on *in vitro *polarized monocyte-derived macrophages. Data are expressed as geometric mean fluorescence intensity (gMFI) of the marker expression after polarization with IFN-γ, IL-4, or IL-10, respectively, divided by the gMFI of the marker expression on unpolarized macrophages. Data are represented as median and interquartile range of at least five independent experiments.

Further to investigate the propensity of monocytes to polarize preferentially toward a specific macrophage subset in SpA, monocytes from SpA and RA patients and healthy donors were polarized *in vitro *in the presence of IFN-γ, IL-4, or IL-10. The expression of specific phenotypic markers was measured with flow cytometry after 4 days of polarization. On spontaneous macrophage maturation in the absence of polarizing cytokines, no differences were noted in the expression of phenotypic markers between SpA and RA patients and healthy donors (data not shown). As previously described in healthy donors [21], IFN-γ specifically upregulated the membrane expression of CD80 and CD64, IL-4 upregulated CD200R and downregulated CD14, whereas IL-10 upregulated CD163 and CD16. After polarization of patient and healthy donor monocytes, we measured a similar modulation of these phenotypic markers in all three groups (Figure 3). In conclusion, we found no evidence for differences in the polarization potential of monocyte-derived macrophages from SpA and RA patients and healthy individuals.

### Intimal lining layer macrophages display a MΦ_IL-10_-like phenotype

**Figure 4:**
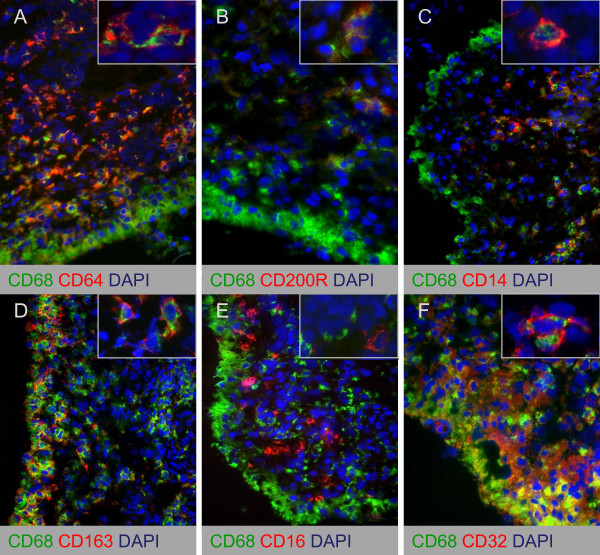
**Double immunofluorescence stainings of CD68 and macrophage polarization markers on synovial tissue from chronic inflammatory arthritis**. Colocalization of CD68 (green) with MΦ_IFN-γ _marker CD64 **(A)**, MΦ_IL-4 _markers CD200R **(B) **and CD14 **(C)**, and MΦ_IL-10 _markers CD163 **(D)**, CD16 **(E)**, and CD32 **(F) **(red) on synovial tissue macrophages. Figures are representative of five spondyloarthritis (SpA) and five rheumatoid arthritis (RA) patients. Higher-magnification photos are included in each figure part.

**Figure 5:**
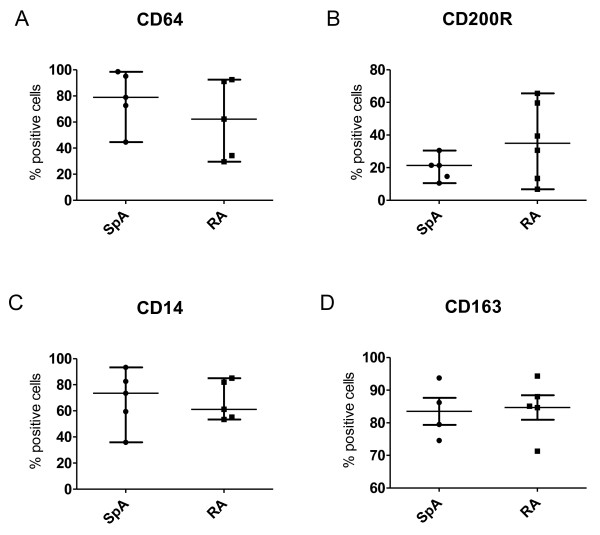
**Expression of polarization markers on CD68^+ ^cells in synovial tissue from chronic inflammatory arthritis**. Figure represents the percentage of CD64^+ ^**(A)**, CD200R^+ ^**(B)**, CD14^+ ^**(C)**, and CD163^+ ^**(D) **cells from the total number of CD68^+ ^cells in five spondyloarthritis (SpA) and five rheumatoid arthritis (RA) patients. Data were acquired by manual quantitative scoring of double-immunofluorescence stainings and are represented as median and interquartile range.

Because we observed an increased expression of CD163, but no differences in the expression of other polarization markers in SpA compared with RA synovitis, we next performed double-immunofluorescence staining to characterize further the polarization phenotype of synovial macrophages. We studied the colocalization of pan-macrophage marker CD68 with MΦ_IFN-γ _marker CD64 (Figure 4A), MΦ_IL-4 _markers CD200R and CD14 (Figure 4B, C), and MΦ_IL-10 _markers CD163, CD16, and CD32 (Figure 4D-F). Intimal lining layer CD68^+ ^cells highly expressed the MΦ_IL-10 _markers CD163 and CD32 (Figure 4D, F), whereas the expression of CD64, CD80, CD200R, CD14, and CD16 was almost absent (Figure 4A-C, 4E) in both SpA and RA. In contrast, synovial sublining macrophages abundantly expressed both the MΦ_IL-10 _markers CD163 and CD32 (Figure 4D, F) and the MΦ_IFN-γ _marker CD64 (Figure 4A). The positive MΦ_IL-4 _marker CD200R showed a low expression and low colocalization with CD68 (Figure 4B), whereas the negative MΦ_IL-4 _marker CD14 strongly colocalized with CD68 (Figure 4C). In agreement with previous reports, CD80 expression was almost absent in synovial tissue (data not shown) [33], whereas CD16 expression was low on CD68^+ ^cells (Figure 4E). Although the number of samples used in these double-immunofluorescence experiments is too small for an accurate statistical analysis, quantification of the staining confirmed the lack of marked differences in the expression of these markers between SpA and RA synovial sublining macrophages (Figure 5). These findings suggest an MΦ_IL-10_-like phenotype of intimal lining layer macrophages in both SpA and RA, whereas synovial sublining macrophages had a more heterogeneous phenotype.

### Synovial sublining macrophages co-express MΦ_INF-γ _and MΦ_IL-10 _markers

**Figure 6:**
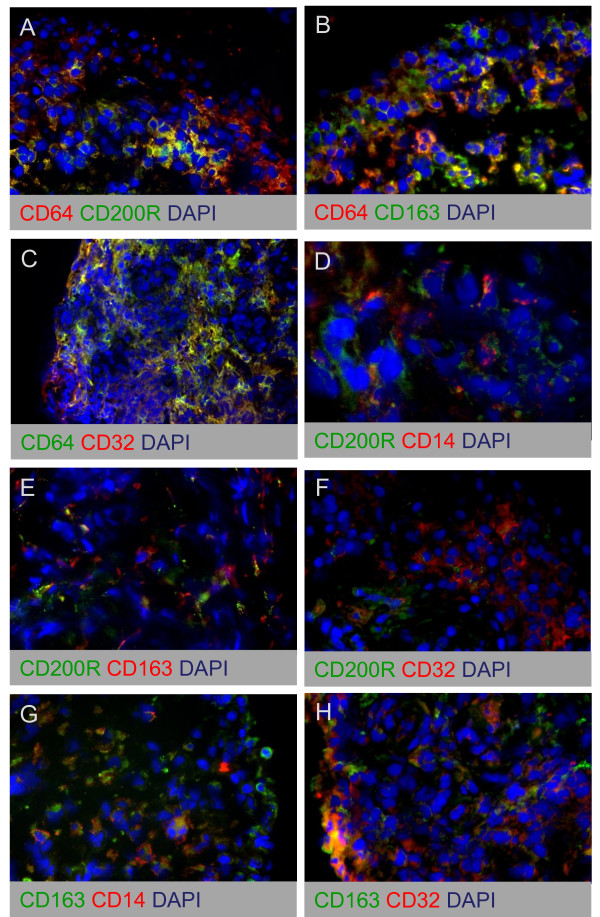
**Double-immunofluorescence stainings of MΦ_IFN-γ_, MΦ_IL-4_, and MΦ_IL-10 _polarization markers on synovial tissue from chronic inflammatory arthritis**. Colocalization of MΦ_IFN-γ _marker CD64 with CD200R **(A)**, CD163 **(B)**, and CD32 **(C)**, colocalization of MΦ_IL-4 _marker CD200R with CD14 **(D)**, CD163 **(E)**, and CD32 **(F)**, and colocalization of MΦ_IL-10 _marker CD163 with CD14 **(G) **and CD32 **(H)**. Figure parts are representative of five spondyloarthritis (SpA) and five rheumatoid arthritis **(**RA) patients.

To characterize further the phenotype of synovial sublining macrophages, we studied the colocalization of MΦ_IFN-γ _marker CD64 with CD200R, CD163, and CD32 (Figure 6A-C), colocalization of MΦ_IL-4 _marker CD200R with CD14, CD163, and CD32 (Figure 6D-F), and colocalization of MΦ_IL-10 _marker CD163 with CD14 and CD32 (Figure 6G, H). These stainings confirmed that both MΦ_IL-10 _markers CD163 and CD32 were expressed on the same cells in SpA and RA synovitis (Figure 6H). However, we also observed a high degree of coexpression between the MΦ_INF-γ _marker CD64 and the MΦ_IL-10 _markers CD163 and CD32 (Figure 6B, C). Furthermore, the smaller macrophage subset that expressed the MΦ_IL-4 _marker CD200R also appeared to coexpress CD64, CD14, CD163, and CD32 (Figure 6D-F). Taken together, these data indicate that synovial sublining macrophages display a mixed MΦ_INF-γ_/MΦ_IL-10 _phenotype. Within this population, a smaller macrophage subset also coexpresses the MΦ_IL-4 _marker CD200R.

## Discussion

Synovial tissue macrophages play a key role in chronic inflammatory arthritis, but the distribution and the function of different macrophage subsets in this process remain largely unknown. Based on the increased CD163 expression in SpA synovitis [3,22-25], we proposed the hypothesis of a preferential M2 polarization in SpA, as opposed to an M1 polarization in RA [27]. The present study aimed to assess this hypothesis experimentally by a detailed characterization of macrophages in inflammatory arthritis, by using a recently validated panel of phenotypic markers for *in vitro *polarized human macrophage subsets [21]. Comparison of macrophage phenotypic marker expression between SpA and RA synovitis confirmed the higher expression of CD163 in the SpA intimal lining layer. Although previous reports showed increased CD163 expression in both the intimal lining layer and synovial sublining, the differences appeared to be larger in the intimal lining layer [3,22].

As CD163 is a prototypical marker for MΦ_IL-10_, we performed three types of experiments to assess whether the increased CD163 expression reflected a preferential M2 polarization in SpA. First, the previously described inverse IFN-γ signature in monocytes from SpA patients [28] suggested that even before entering the synovial compartment, myeloid cells may be skewed to polarize toward M2 rather than M1. Our analysis of peripheral blood monocytes, however, did not reveal phenotypic differences between SpA, RA, and healthy controls. In humans, three monocyte subsets have been identified: classic CD14^+^CD16^-^, intermediate CD14^+^CD16^+^, and nonclassic CD14^dim^CD16^+ ^monocytes. Among these subsets, CD14^+^CD16^+ ^monocytes were described to be recruited in tissues during inflammation and to produce high amounts of TNF [32,34-37]. Although other publications report contrasting findings of either an increased [38-40] or a decreased [41] percentage of CD14^+^CD16^+ ^monocytes in inflammatory compared with healthy conditions, our experiments did not identify differences in these monocyte subsets between SpA and RA patients and healthy donors. Additionally, we did not observe differential expression of other phenotypic markers, such as CD64, CD16, and CD32, whereas the expression of CD80 and CD163 was very low on peripheral monocytes, as previously described [42,43].

Second, we assessed whether a potential bias toward M2 may appear during the differentiation of monocytes toward macrophages. *In vitro *polarization experiments with monocyte-derived macrophages failed, however, to indicate preferential polarization to a distinct subset in SpA compared with RA patients and healthy donors. These data suggest that the CD163^+ ^macrophage phenotype in SpA synovitis is determined by the local inflammatory milieu rather than by intrinsic myeloid alterations. Previous publications showing CD163 upregulation during *in vitro *macrophage maturation in the presence of SpA synovial fluid [27] and a strong synovial CD163 downregulation on effective antiinflammatory treatment [12,13] support this observation.

Third, we assessed whether the local upregulation of CD163 on intimal lining layer macrophages in SpA was associated with differential expression of other phenotypic polarization markers between SpA and RA. This was not the case, as, for example, both the MΦ_IL-10 _marker CD32 and the prototypical MΦ_IFN-γ _marker CD64 were similarly expressed in both diseases. These data question whether the altered expression of CD163, which is a reliable M2 marker *in vitro*, necessarily reflects a marked M2 versus M1 polarization *in vivo*. Supporting the notion that the phenotype of macrophage subsets *in vivo *is more complex than the conceptual *in vitro *framework of M1 and M2, detailed double-staining analysis showed that synovial sublining macrophages display a mixed phenotypic profile with a high colocalization of MΦ_IFN-γ _and MΦ_IL-10 _markers. Similar observations of mixed polarization profiles were already described for human adipose tissue macrophages (ATMs), which show features of both alternative (CD206, CD163, IL-10, TGF-β) and classic (TNF, IL-6, IL-23, IL-8) activation [44,45]. An important question raised by this *in vivo *complexity is whether these cells exert both the pro- and antiinflammatory functions, which have been attributed to M1 and M2 macrophages, respectively, or whether they are steady-state cells waiting for additional signals to determine their ultimate phenotype and function. The latter hypothesis implies a large plasticity of tissue macrophages, as previously suggested in tumor environment [46], adipose tissue [47], and atherosclerosis [48]. Tumor-associated macrophages were described to have M2 properties, with low inflammatory chemokine receptors, poor antigen presentation, high IL-10, and low IL-12 production [49-53]. Interestingly, polarization toward an M1-like phenotype was shown to suppress tumor progression [54]. Adipose tissue macrophages were also reported to switch from an M2-like phenotype in normal adipose tissue to an M1-like phenotype in diet-induced obesity [55,56], but reports exist of a predominant M2 activation in insulin resistance and obesity [57]. The same holds true for the macrophages in the vascular wall, which were described to switch from an M2- to an M1-like phenotype and become so-called foam cells [58-60], whereas other authors describe an accumulation of M2-like cells in the atherosclerotic plaque [61]. Although the interpretation of these studies remains difficult in the absence of single markers that can unequivocally discriminate between macrophage subsets *in vivo *[15], the macrophage phenotype in the synovial sublining may reflect the same plasticity as in tumors, fat tissue, and atherosclerosis.

In contrast with the mixed phenotype of synovial sublining macrophages, the intimal lining layer macrophages clearly displayed an MΦ_IL-10_-like phenotype. We observed the same differences between the phenotype of intimal lining layer and synovial sublining macrophages, not only in SpA and RA but also in gout synovitis (data not shown), suggesting that this macrophage distribution pattern is a global feature of synovial inflammation. Together with the low expression of CD14 in the intimal lining layer, this observation may fit a model according to which the intimal lining layer contains mainly mature resident macrophages, whereas the synovial sublining is actively infiltrated with immature monocyte-derived macrophages. This model is in agreement with publications showing that the number of synovial sublining but not intimal lining layer macrophages is associated with disease activity in RA and correlates with response to therapy [7-9].

Concerning the differences between SpA and RA, this model would predict that the synovial sublining is similarly infiltrated by monocyte-derived macrophages with a mixed phenotype in both pathologies. However, the MRP8/14^+ ^infiltrating macrophages were shown to accumulate highly in the intimal lining layer in RA [3] and secrete a variety of proinflammatory mediators [62]. In contrast, the SpA intimal lining layer appears to consist mainly of resident, MΦ_IL-10_-like macrophages, which is in accordance with the lower levels of M1-derived cytokines in SpA compared with RA synovial fluid [27] and with the less-destructive appearance of SpA synovitis.

Based on these findings, modulating the polarization and/or the migration of distinct macrophage subsets to the intimal lining layer would represent interesting therapeutic approaches for chronic synovitis.

## Abbreviations

CD32: cluster of differentiation 32; CD163: cluster of differentiation 163; FCS: fetal calf serum; gMFI: geometric mean fluorescence intensity; IMDM: Iscove Modified Dulbecco Medium; RA: rheumatoid arthritis; SpA: spondyloarthritis.

## Competing interests

The authors declare that they have no competing interests.

## Authors' contributions

CAA, PPT, and DLPB participated in the design of the study. Acquisition of the data was performed by CAA, TN, and MJH. Data were analyzed and interpreted by CAA and TN. The manuscript was drafted by CAA and was revised by MJH, PPT, and DLPB. All authors read and approved the final manuscript.
